# PM2.5 Pollution Decrease in Paris, France, for the 2013–2024 Period: An Evaluation of the Local Source Contributions by Subtracting the Effect of Wind Speed

**DOI:** 10.3390/s25216566

**Published:** 2025-10-24

**Authors:** Jean-Baptiste Renard, Jérémy Surcin

**Affiliations:** 1 Laboratoire de Physique et Chimie de l’Environnement et de l’Espace, F-45071 Orléans, France; 2 Pollutrack Inc., F-75020 Paris, France

**Keywords:** PM2.5 mass-concentration, pollution, Paris, trend, wind speed

## Abstract

**Highlights:**

**What are the main findings:**

- A direct link is highlighted between PM2.5 mass-concentrations and wind speed in Paris, France.
- The PM2.5 levels are decreasing with increasing wind speed, up to an inflection point at 6 m·s^−1^ from which the PM2.5 levels remain almost constant.

**What is the implication of the main finding:**

- The trend for PM2.5 mass-concentration decrease can be estimated by subtracting the effect of wind speed.
- The PM2.5 background contribution due to local emission sources can be estimated to be of around 4% per year in Paris.

**Abstract:**

Measuring the long-term trend of PM2.5 mass-concentration in urban environments is essential as it has a direct impact on human health. PM2.5 levels depend not only on the intensity of local emission sources and on imported pollution, but also on meteorological conditions (e.g., anticyclonic versus windy conditions), which leads to yearly variations in mean PM2.5 values. Two datasets available for Paris, France, are considered: measurements from Airparif air quality agency network and from the Pollutrack network of mobile car-based sensors. Also, meteorological parameters coming from ERA5 analysis (ECMWF) are considered. Annual values are calculated using three different statistical methods, which yield different results. For the 2013–2024 period, a clear relationship between wind speed and PM2.5 mass-concentration levels is established. The results show a linear decrease in both concentration and standard deviation for wind speeds in the 0–6 m·s^−1^ range, followed by nearly stable values for wind speed above 6 m·s^−1^. This behavior is explained by the dispersive effect of strong winds on air pollution. Under such conditions, which occur about 10% of the time in Paris, the contribution of persistent background sources can be isolated. Using the 6 m·s^−1^ threshold, the average annual linear decrease in emissions from local sources is estimated at 4.1 and 4.3% per year for the Airparif and Pollutrack data, respectively. Since 2023, the annual background value attributed to emission has been close to 5 µg·m^−3^, in agreement with WHO recommendations. This approach could be used to monitor the effects of regulations on traffic and heating emissions and could be applied to other cities for estimating background pollution levels. Finally, future studies should therefore prioritize number concentrations and size distributions, rather than mass-concentrations.

## 1. Introduction

Accurate measurements of PM2.5 in urban conditions are essential, as these particles have a direct effect on human health [1,2,3,4,5]. The smallest particles are by far the most dangerous, since they can penetrate deeply into the body and affect multiple organs. They can trigger asthma attacks, respiratory diseases (including COVID-19), cancers, heart attacks, strokes, neurological disorders, alteration of placenta, worsening diseases morbidity, and thus increasing the mortality rate among the population living in polluted areas [6,7,8,9,10,11,12,13].

Some of these particles, called primary particles, are directly produced by anthropogenic activities [14,15], mainly traffic, constructions and wood- or fossil fuels heating, producing primary Particular Matter (PM). Secondary particles, on the other hand, result from complex chemical reactions involving sunlight, ammonium (from agricultural activities), nitrogen oxides, and sulfur oxides (from traffic and industrial activities). In urban areas, such as for the French capital city, Paris, the main sources are often attributed to traffic and heating, but agricultural activities during spreading periods also play a significant role [16].

In September 2021, the World Health Organization (WHO) recommended PM2.5 mass-concentrations (integrated mass of all particles smaller than 2.5 µm) limits of an annual mean below 5 µg·m^−3^ and a 24 h mean below 15 µg·m^−3^; this daily limit should not be exceeded for more than 4 days per year [17]. The revised European Ambient Air Quality Directive, released in 2024 [18], aligns the 2030 European Union air quality standards more closely with WHO recommendations, and confirms the importance of studying PM2.5 (all particles smaller than 2.5 µm) to establish a long-term trend in PM evolution. Studies must focus on urban zones, which are often the most polluted and where most of the population lives.

Among the European large cities, Paris, France, ranks among the most polluted [19]. Paris has 2.2 million inhabitants, and about 10 million inhabitants including suburbs. It also has one of the highest population densities in Western Europe as it is a very compact city (105 km^2^). Situated in a geographical basin, Paris is poorly ventilated.

Paris and its suburbs are characterized by relatively few polluting industries, but intense traffic and nearby intensive agricultural activities significantly affect air quality. Regional urban background conditions can be strongly influenced by long-distance transport of pollutants, which may account for up to 70% of the PM2.5 mass-concentration [20,21,22,23,24,25,26]. In addition, Paris is circled by a motorway ring road with traffic volume of up to 1,200,000 vehicles per day [27]. Thus, local primary PM2.5 sources are mainly car traffic (diesel exhaust, tire and break wear, and asphalt abrasion), and residential heating in winter [28]. These sources are responsible for major pollution peaks under anticyclonic conditions. The higher levels are measured around the ring road [29]. Within the city, mean concentrations are higher in the northeast than in the southwest, mainly due to the transport of air mass driven by urban topography and a combination of broad avenues and canyon streets [30]. As a result, the spatial distribution of PM mass-concentrations can vary significantly within just a few hundred meters, with persistent hot-spots closely linked to traffic [31].

PM2.5 mass-concentration trends in most European cities show a decrease of about 2.5% per year, mainly due to stricter vehicle emission regulations [32,33]. For Paris, the official air quality monitoring network (Airparif) reports that PM2.5 mass-concentration has decreased by 55% between 2005 and 2024, and by 35% between 2015 and 2025 [34]. These values were obtained without accounting for meteorological variability, even though the frequency of anticyclonic versus windy conditions varies greatly from year to year. Such variability can introduce fluctuations and bias in the curve describing the long-term evolution of PM2.5.

Meteorological conditions are indeed a major driver of PM pollution levels, particularly wind strength [35,36,37,38,39,40]. Under windy conditions, locally produced pollution is dispersed and diluted. Rainfall can wash out the boundary layer, temporarily reducing PM concentrations, but levels generally return to normal within several hours [41]. During anticyclonic conditions, local sources increase both primary and secondary PM levels, as pollutants emitted in previous days accumulate due to the lack of wind. When winds eventually return, the pollution dissipates. This description is, of course, simplified: imported pollution from industrial activities, major fires, or desert dust episodes—sometimes transported over hundreds or even thousands of kilometers—must also be considered, although their contribution can vary greatly [42,43,44,45].

It therefore seems necessary to correct this curve for the effects of the wind strength, in order to remove the oscillations and determine the mass-concentration decrease more precisely. In this study, we propose a method to determine the PM source intensity, using hourly wind speed data and two different datasets of PM2.5 mass-concentrations in Paris, although other weather parameters could also influence PM2.5 levels and contribute to the dispersion of their values. The aim of this paper is not to evaluate the effect of all the weather parameters on PM2.5 levels, since this could be a complex problem with multiple solutions. For example, humidity close to 100% may indicate rain, storms, or fog, each producing very different PM2.5 levels. This paper will focus solely on evaluating the effect of wind speed on PM2.5 levels.

## 2. Materials and Methods

The first set of pollution data originates from Airparif network, the official air quality measurements of PM2.5 mass-concentrations [46]. These were obtained using instruments (microbalances or Beta-attenuation mass monitors) that provide data meeting the quality objectives defined in the evaluation protocol MO-1347, and compliant with the data quality objectives (uncertainty, data capture) for normative measurement, as described in the European Commission Directive 2008/50/EC [47]. The data are available on an hourly basis but must be averaged to daily values in order to comply with the legal requirements regarding pollution thresholds. Since the operating cost of measuring stations is high, only a limited number of fixed stations are available inside Paris, increasing from 3 stations in 2012 to 6 stations in 2024. When including the suburbs near Paris, these numbers rise to 8 and 14, respectively. The stations are strategically located in different urban environments, both close to the traffic and in background conditions, to capture the range of urban and peri-urban configurations. Although these measurements are highly accurate, they do not fully represent the spatial heterogeneity of pollution across the whole city.

For the present study, all Airparif data are averaged at both hourly and daily scales to estimate the mean PM2.5 mass-concentrations. Some bias may occur when averaging, particularly when pollution hot-spots that can vary spatially and temporally are not considered. In this study, we consider data for a 12-year period (2013–2024) for which consistent data are available for both PM2.5 mass-concentrations and meteorological parameters.

The second source of pollution data, available for the 2018–2024 period in Paris, comes from the mobile Pollutrack sensors installed on up to 500 rooftops of (mainly) electric vehicles belonging to Enedis and Geopost companies [29,31]. These cars circulate mostly within Paris, and the less frequent measurements from the suburbs are not considered in this analysis. The sensors are oriented in the opposite direction of travel, ensuring a more stable airflow for relative speeds up to about 40 km·h^−1^.

The Pollutrack sensors consist of a compact optical particle counter in which the particles pass through a laser beam inside an optical chamber. Particle number concentrations (number of particles of a given size) in the 0.5–2.5 µm size range are accurately measured and converted into PM2.5 mass-concentrations using internal calibrations. Since particle mass density is sensitive to relative humidity [48], a first order correction is applied during high-humidity episodes, with humidity data retrieved in real time from local weather stations. For a given size, wet particles are often lighter than dry particles, as water density is lower than that of minerals or carbonaceous particles. All Pollutrack data in Paris are also averaged hourly and daily to match the lower temporal resolution of Airparif data. Thanks to their broader spatial coverage, these data are expected to better capture the local heterogeneity of pollution. An intensive validation program was carried out by collocating some Pollutrack sensors with the Airparif stations at 3 locations in the Paris region. A mean difference of 0.1 ± 3.5 µg·m^−3^ was observed over several months of comparison [29]. Thus, the two datasets are consistent when considering a large number of measurements, as performed in this paper, and can be used together to analyze PM2.5 temporal trends.

**Figure 1:**
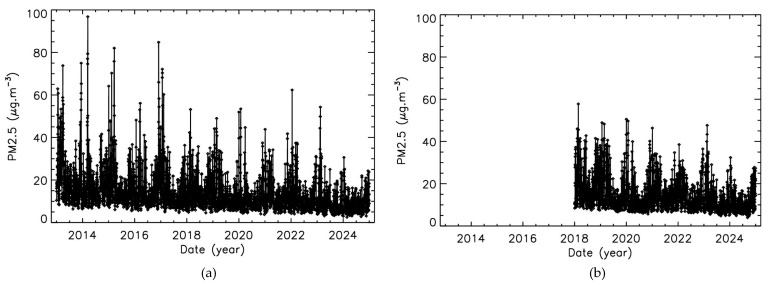
Time-evolution of daily PM2.5 mass-concentrations; (**a**): Airparif; (**b**): Pollutrack.

For each dataset, hourly data were integrated to produce the time-evolution of daily PM2.5 mass-concentration values shown in Figure 1. Although some differences can be observed in the maximum pollution levels, due to the different number of sensors and the resulting mean values, the two datasets display similar behavior. First, a clear annual cycle is evident, with higher pollution peaks during winter due to the additional contribution of heating to traffic emissions, but also to the lower altitude of the upper limit of the boundary layer [49]. Since the boundary layer height is lower in winter than in summer, the vertical dispersion of particulate matter is reduced, leading to higher concentrations near the surface. Second, a long-term decreasing trend is apparent, despite interannual variability in maximum mass-concentrations, likely linked to weather fluctuations.

**Figure 2:**
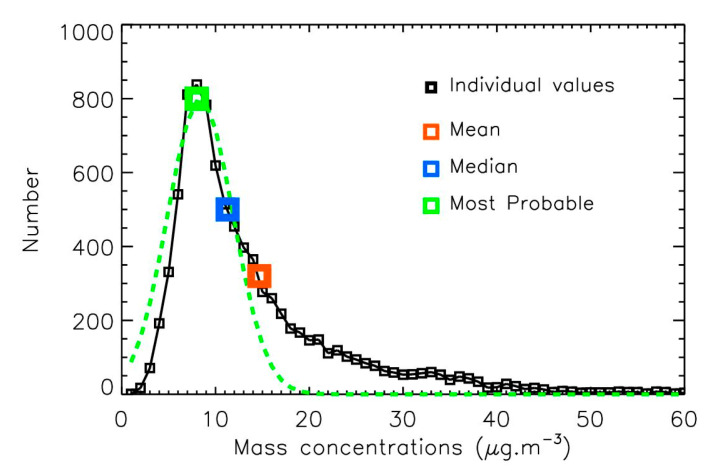
Histogram of the hourly 2017 Airparif PM2.5 mass-concentrations, and yearly value obtained with 3 different methods: mean calculation, median calculation, and most probable value obtained from a Gaussian fit (dashed green line).

The histogram of daily mass-concentrations does not perfectly follow a log-normal distribution, as shown in Figure 2 for the Airparif data in 2017. The number distribution rises sharply to reach a maximum at 8 µg·m^−3^, and then gradually decreases, extending up to 50 µg·m^−3^. This suggests that a simple mean calculation may not be fully appropriate for determining the annual PM2.5 value, since both the width and the peak of the number distribution can vary from one year to another year depending on weather conditions.

Among the various statistic methods available to establish yearly PM2.5 trend calculations, we propose 3 approaches. The first is the widely used annual mean of all hourly or daily values, despite the limitation presented above. The second is the annual median of all hourly or daily values. The third consists of estimating the most probable concentration per year by fitting a Gaussian curve to the mass-concentration histogram (here by step of 1 µg·m^−3^) and identifying the peak value. The mean value method is sensitive to high pollution peaks and therefore tends to yield higher yearly estimates than the other two methods. By contrast, the most probable value method is dominated by the persistent background levels. Figure 2 presents the results for 2017; a factor of about two is observed between the mean and the most probable value, which is a significant difference. Consequently, in the following analysis, we apply all three methods that can reflect different aspects of the pollution value distribution to calculate the annual PM2.5 trend.

Finally, to correlate PM2.5 mass-concentrations with weather conditions, several meteorological stations in Paris region could be considered. However, as for PM pollution, averaging very local measurements carries the risk of bias due to urban topography and may not always reflect mean values. Therefore, we use hourly weather data coming from ERA5 analysis at a height of 10 m, which provides spatially averaged values over Paris for wind speed and direction, temperature, and humidity. This database is produced by the ECMWF (European Meteorological forecast) as part of the Copernicus Earth Observation Program of the European Union.

## 3. Results

### 3.1. Uncorrected PM2.5 Trends

**Figure 3:**
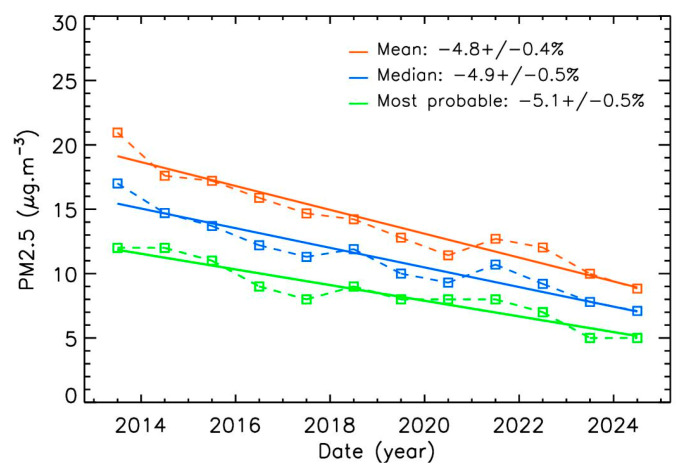
Annual evolution of Airparif PM2.5 mass-concentrations with three different methods of calculation, and associated decrease percentages per year.

Using a linear fit for all annual PM2.5 concentration values, as is commonly performed in this kind of analysis, the trend can be calculated as the slope of the fit divided by its first value. The error bars are calculated as the error on the slope divided by the first value of the fit. Therefore, the mean decrease obtained over 12 years of Airparif measurements, based on the three calculation methods for the yearly values, fall in the range of 4.8–5.1% per year (Figure 3, and Table 1 at the end of part 3). The correlation between the experimental data and linear fit is 0.95 for Airparif and ranges from 0.76 to 0.89 range for Pollutrack (with a smaller number of data points), confirming that the proposed trends are robust.

**Figure 4:**
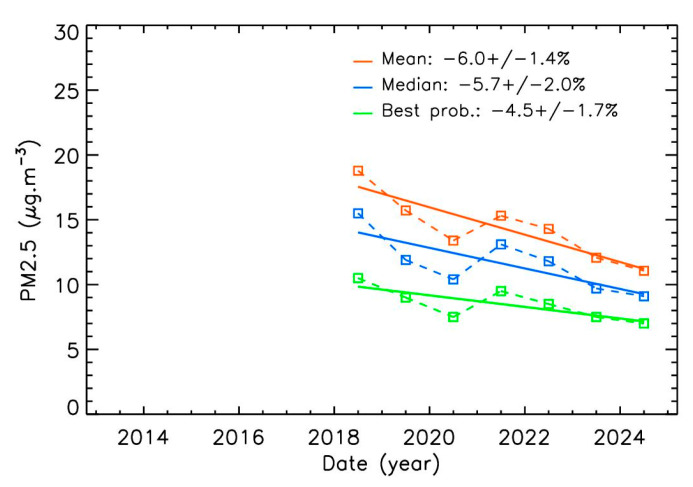
Annual evolution of Pollutrack PM2.5 mass-concentrations with three different methods of calculation, and associated decrease percentages per year. The mean uncertainty, compared to Airparif measurements, is 0.1 µg·m^−3^.

This is greater than the 2.5% mean decrease reported for most European cities [43,44]. It is also greater than the 3.5% per year estimated by Airparif for the broader Paris region, which includes surrounding rural areas where traffic-related emissions are lower. Slightly higher values are obtained using the Pollutrack dataset (Figure 4), with mean decreasing values in the range of 4.5–6.0%, although over a shorter period. Year-to-year values oscillate around the trend line, likely due to variations in weather conditions (e.g., windy and rainy periods versus stable anticyclonic conditions).

**Figure 5:**
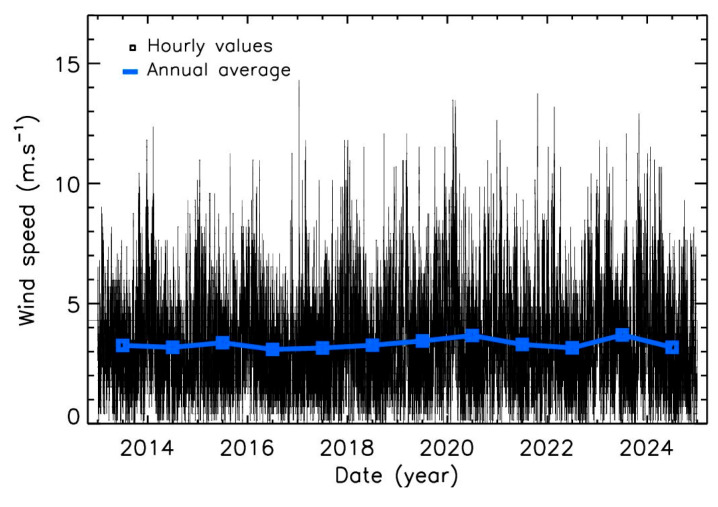
Hourly and average annual value for wind speed in Paris, at a height of 10 m.

It can be expected that wind speed is the main parameter influencing PM2.5 levels. Therefore, a detailed analysis of mean wind speed over Paris was conducted. Figure 5 shows the hourly wind speed values at 10 m above the ground level, along with annual averages over the 12-year period. Overall, winds are stronger in winter than in summer, although strong day-to-day variability is observed. Most wind speeds are below 6 m·s^−1^, but between 6% and 13% of the values exceed this threshold, depending on the year, corresponding to a cumulative period of 22 to 47 days per year. Significant interannual variability is also present. For example, winds were stronger than usual for several weeks during the winters of 2019/2020 and 2023/2024, whereas unusually low values persisted for several months in mid- and end-2022 (thus significantly increasing the overall 2022 mortality, exceeding the 2020 and 2021 COVID-19 years). A variability of about 20% in the average wind speed is detected, highlighting the importance of considering weather variability when analyzing PM2.5 trends.

### 3.2. Effect of Wind on PM2.5 Mass-Concentration Levels

In urban environments or industrial zones with continuous activities, it can be first assumed that when the wind speeds are high, PM2.5 produced by local sources are efficiently dispersed. Nevertheless, PM2.5 mass-concentrations are constantly replenished by these sources, and an equilibrium may be reached between production and dispersion, resembling a steady-state regime. This balance can, however, be disturbed by stormy conditions with rain and strong winds, which can sharply reduce PM2.5 concentrations. Also, imported pollution can increase PM2.5 levels. Conversely, when winds speed is too low, local emissions cannot be adequately dispersed, leading to higher pollution levels. If such anticyclonic conditions persist, PM2.5 levels will continue to rise day after day, as new contributions accumulate to the previous ones.

**Figure 6:**
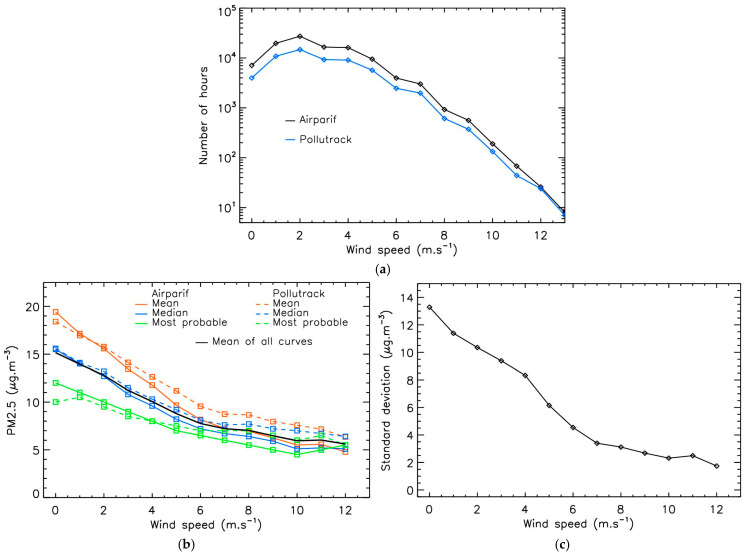
Evolution of PM2.5 mass-concentrations with wind speed in Paris for the 2013–2024 period. (**a**): Number of hours available for the analysis; (**b**): Evolution of PM2.5 mass-concentrations with wind speed; (**c**): Standard deviation of Airparif PM2.5 mass-concentrations calculated for each wind speed bin.

To evaluate these effects, the 12-year hourly Airparif PM2.5 mass-concentrations were averaged for each wind value in the 0–12 m·s^−1^ range 1 m·s^−1^ bins. The number of data points follows a log-normal distribution for both Airparif and Pollutrack datasets, with a regular decrease in sample size along increasing wind speed after 2 m·s^−1^ (Figure 6a). As a consequence, there are too few hourly values above 12 m·s^−1^ to provide statistically significant results.

Figure 6b shows the evolution of PM2.5 mass-concentration with wind speed for both datasets. The same trend is observed: PM2.5 mass-concentrations decrease as wind speed increases. As expected, the curve for the most probable value is below those of the mean and median values, although all curves exhibit the same overall behavior. First, mass-concentrations decrease almost linearly when wind speed increases from 0 to 6 m·s^−1^. Then, an inflection point appears at 6 m·s^−1^. Finally, for higher wind speeds, the mass-concentrations decrease only slightly. Similarly, the standard deviation of PM2.5 mass-concentrations calculated for each wind speed bin decreases from 0 to 6 m·s^−1^ and remains stable after 6 m·s^−1^ (Figure 6c). These results are not correlated with the number of hours used for the analysis shown in Figure 6a, thus strengthening confidence in their statistical significance.

The decreasing standard deviation values with increasing wind speed clearly illustrates not only the role of wind in dispersing PM2.5, but also the cumulative effect of prolonged low-wind conditions on pollution levels. Each new consecutive day with low winds increases PM2.5 mass-concentrations compared to the previous day. Under such conditions, the PM2.5 levels remain elevated for several consecutive days rather than for just a single day of anticyclonic conditions, even if the local sources or pollution are the same. Conversely, above the 6 m·s^−1^ threshold, dispersion by winds dominates over local emissions, resulting in a quasi-stable regime on the PM2.5 mass-concentrations. This regime is not perfectly stable, as concentrations seem to continue to decrease slightly with increasing wind speed. This decrease falls within the uncertainties of the instruments at low concentration levels of a few µg·m^−3^, but the possibility of systematic errors cannot be excluded, such as the effect of pollutant transport from distant sources.

These results show that a direct correlation between PM2.5 and wind time series cannot be established, since the consecutive number of anticyclonic days could affect the absolute value of the PM2.5 mass-concentrations and thus the correlation. Nevertheless, this 6 m·s^−1^ cutoff is empirically determined and just for Paris. This value should be corroborated by future studies that consider measurements of all possible sources despite their large number and spatial variability, as well as from atmospheric transport theory considering the city’s specific topography. Such modeling works on transport are still in progress for the city of Paris [50,51,52].

The PM2.5 mass-concentrations observed at and above this inflection point can therefore be considered a good estimate of the contribution from permanent sources in Paris. The 6 m·s^−1^ threshold thus provides a useful criterion to distinguish between permanent background values and pollution peaks associated with anticyclonic conditions that can vary considerably from one year to another year.

By contrast, no significant correlation between PM2.5 mass-concentrations is observed with the other meteorological parameters (wind direction, humidity, temperature) beyond the usual seasonal ones. For instance, during winter anticyclonic conditions with negative temperatures and shallow boundary layer, stronger increases in PM2.5 mass-concentrations can occur. However, pollution peaks may also appear under warmer anticyclonic conditions in other seasons. Similarly, high humidity values can be associated with very different weather conditions, such as haze, fog, rain, or storm, and thus correspond to different wind regimes. Finally, the absence of correlation with wind direction could be due to the fact that the main source of PM2.5 pollution comes from the ring road that encircles Paris. Overall, wind speed appears to be the most relevant meteorological parameter associated with PM2.5 pollution.

### 3.3. Time-Evolution PM2.5 Mass-Concentrations During Strong Winds

The PM2.5 mass-concentrations trend for the 2013–2002 period can now be evaluated for two configurations: winds below 6 m·s^−1^ and winds above 6 m·s^−1^, as performed in part 3.1. The correlation between the experimental data and linear fit ranges from 0.94 to 0.96 for Airparif and ranges from 0.74 to 0.91 range for Pollutrack (with a smaller number of data points), confirming that the proposed trends are robust.

**Figure 7:**
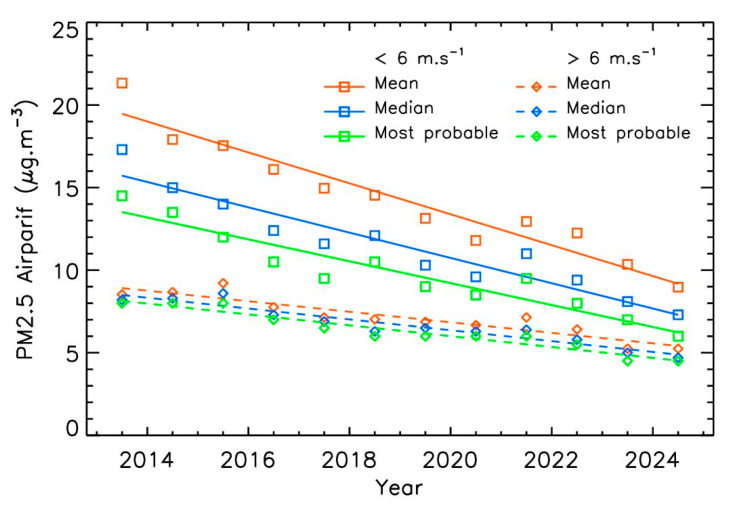
Annual Airparif PM2.5 trends for two different wind ranges, using different statistical calculations.

The results for the Airparif dataset (Figure 7 and Table 1) show that the mean trend when winds are below 6 m·s^−1^ is of −4.8 ± 0.5% for the three calculation methods, which is consistent with the overall trend obtained without wind filtering (−4.9 ± 0.5%, Figure 3). In contrast, the trend for winds above 6 m·s^−1^ is significantly lower, at 4.1 ± 0.4% per year. The main effect of this wind speed selection lies in the absolute mass-concentrations values, which are about twice as low under stronger wind conditions than under low-wind conditions. In the case of strong wind conditions, the trends derived from the three methods are very close to each other, indicating that the yearly PM2.5 distribution under strong winds follows a log-normal distribution.

**Figure 8:**
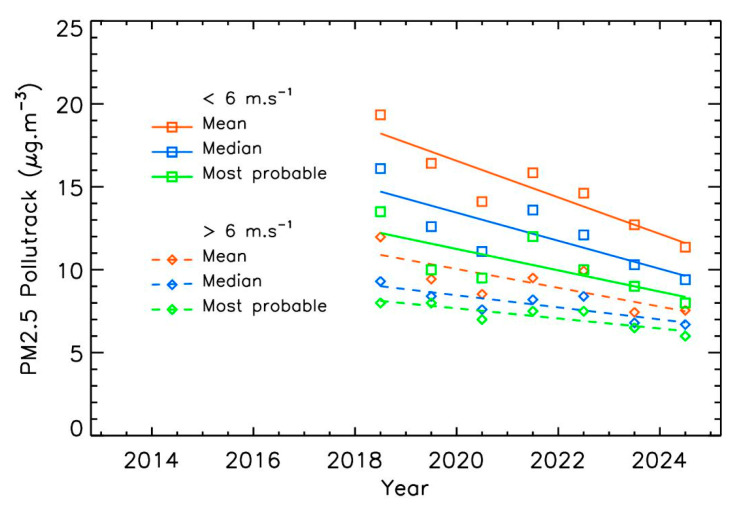
Annual Pollutrack PM2.5 trends for two different wind ranges, using different statistical calculations. The mean uncertainty, compared to the Airparif measurements, is 0.1 µg·m^−3^.

Similar results are obtained with the Pollutrack dataset for winds above 6 m·s^−1^ (Figure 8 and Table 1). Although these data offer better spatial coverage within Paris, they cover a shorter time period, making trends more difficult to calculate precisely. Nevertheless, the consistency between the two datasets obtained with different instrumental techniques strengthens confidence in the analysis. As with Airparif, the mean trend for winds above 6 m·s^−1^ (–4.3 ± 0.4% per year across the three methods) is lower than that for weaker winds (–5.7 ± 1.7% per year). However, the Pollutrack data are moderately scattered and cover a limited time period, highlighting the limitations of applying a simple linear fit in this case. As for the Airparif, PM2.5 mass-concentrations under strong winds are about twice as low as under weaker winds. Finally, the error bar for the trend calculated using the most probable yearly values is significantly reduced in the Pollutrack dataset for winds above 6 m·s^−1^, suggesting that this statistical method may be particularly suitable for further analyses.

All these results could argue for a revision of the PM2.5 pollution trends and of the contribution of PM2.5 sources in Paris. This is discussed in the ensuing section.

## 4. Discussion

Due to their effect on human health, the PM2.5 mass-concentration levels must be accurately measured in major cities such as Paris. To comply with the new European Ambient Air Quality Directive [18], the sources of PM2.5 emissions must be accurately identified and controlled. The ongoing effort to reduce PM2.5 emissions focuses on ameliorating thermic motor exhausts, reducing traffic and the number of vehicles within Paris, and better controlling wood-heating.

The decreasing trends commonly reported for PM2.5 mass-concentrations in Paris are based on mean values [46], thus providing an estimate of the average pollution level to which citizens are exposed. However, such values are dominated by strong pollution peaks often associated with meteorological conditions that can persist from several days to weeks. Due to this variability, some annual averages may appear better than others, complicating trend estimation over short periods. This makes it difficult to obtain statistically robust results when the authorities wish to rapidly assess the effects of emission-reduction measures, such as restrictions on road traffic. Furthermore, mean values alone do not capture the heterogeneity of PM2.5 levels within a city. Maps showing the number of days above a given threshold at local scales could be a better way to evaluate the real exposure of citizens to air pollution [29], making it necessary to couple as many measurements and modeling outputs as possible.

Simple mean calculations are also insufficient to accurately estimate the long-term evolution of permanent or recurrent source emissions These sources provide the background conditions and the daily minimum PM2.5 concentrations to which the citizens are continuously exposed. Under low-wind conditions, these concentrations accumulate from one day to the next if dispersion does not occur. By contrast, PM2.5 mass-concentrations observed during strong winds can be considered, first of all, as an indicator of the level of persistent sources of pollution. These sources include local traffic and heating (both primary and secondary aerosols) but also imported pollution from industrial and agricultural activities from other regions or countries [19,20] during low or moderate winds not strong enough to disperse their contribution. Of course, this analysis is limited to surface-level pollution and to the lower boundary layer, and does not account for particle plumes transported at higher altitudes that may occasionally descend.

In Paris, this background permanent pollution can be estimated from measurements during the ~10% of time where the winds exceed 6 m·s^−1^. Source emissions have decreased from ~9 to ~5 µm.m^−3^ over the past 12 years, representing a ~45% reduction. This value lies between the 35% and 55% decrease previously reported [46], depending on the chosen time period considered, but without accounting for the variability of anticyclonic conditions. The trend established here appears linear, probably the steady reduction in road traffic (−51% in Paris between 2002 and 2022 [53]). Traffic-related emissions are likely to continue decreasing in the coming years, but maybe more slowly. Although essential vehicle activity will remain, and even with electrification, non-exhaust emissions from brakes, tires, and resuspension [54,55] will sustain a baseline level of PM2.5 mass-concentrations. The decrease also reflects stricter regulations of engine emissions, especially diesel, as well as restriction on wood-burning PM emissions, in line with observations from other regions worldwide [56,57,58].

For 2023–2024, background levels in Paris are close to the WHO annual guideline of 5 µg·m^−3^. This result suggests that the level of emissions sources is now quite well reduced. In an ideal scenario with no anticyclonic events and no imported pollution, citizens could be exposed to an acceptable PM2.5 level.

Nevertheless, these optimistic results must be tempered by three important points. First, the number of days with high pollution levels due to anticyclonic conditions varies from year to year, as does the duration of consecutive low-wind periods, and such episodes can seriously affect public health even if the background levels presented above are low. Second, the reported trend reflects citywide averages, without accounting for the significant spatial variability. Areas near major roads can experience concentrations up to twice those in quieter areas [29], and may never reach such favorable levels. Finally, most of the studies, including this one, focus on mass-concentrations as references, which are dominated by the largest particles, while the most harmful particles are those smaller than 1 µm, and particularly ultrafine particles smaller than 0.1 µm [59].

Future studies should therefore prioritize number concentrations and size distributions, rather than mass-concentrations that aggregate the contribution of all particles below a cutoff size (here 2.5 µm). Such measurements require expensive instruments, usually operated only during specific campaigns and rarely on a continuous basis. However, since late 2019, Airparif has permanently operated such an instrument in central Paris. Although the data are not yet publicly available, analyses covering October 2019 to December 2022 reported a mean concentration of 8100 ± 4800 particles·cm^−3^ (8–100 nm), with peaks close to 40,000 particles·cm^−3^ [60]. This study confirmed that road traffic is the main source of ultrafine particles, highlighting the importance of such measurements for public health and future air quality regulations.

## 5. Conclusions

A statistical method based on the wind speed, using a cutoff at 6 m·s^−1^, made it possible to distinguish, in Paris, between permanent background pollution during high wind speeds and pollution peaks occurring under anticyclonic conditions with low wind speeds. With this approach, the contribution of permanent anthropogenic sources can be evaluated, as well as their time-evolution, without being biased by the variability in the number and duration of anticyclonic episodes. This method should therefore be recommended for assessing the real impact of the new local regulations targeting traffic, engine and exhaust emissions, and wood-burning. Thanks to these regulations, PM2.5 mass-concentrations in Paris have decreased linearly by −4% per year over the last 12 years. Using the most optimistic estimates, background PM2.5 levels since 2023 are close to the WHO annual guideline of 5 µg·m^−3^. Nevertheless, it must be remembered that during strong anticyclonic events, pollution can rise sharply over several days or weeks, significantly affecting both the observed mean decrease and public health.

This study was conducted for a single city with a specific geophysical context; Paris, is surrounded by hills that limit pollutant dispersion. Future studies should extend this approach to other cities with different topographies to verify whether background conditions and sources contributions can indeed be retrieved using a wind speed cutoff. To ensure the consistency of the results, it is recommended to use at least two independent sets of measurements, in order to verify that the results obtained from the limited number of air quality agency monitoring stations are truly representative of the real PM2.5 pollution levels. It will then be possible to estimate if the same cutoff applies everywhere, or whether it varies upon local topography such as in coastal cities, continental cities, windy cities, or mountainous regions. Considering such approach, two different values should be produced for each city to better characterize the PM2.5 pollution levels and, therefore, its evolution over time: the annual mean value calculated under all weather conditions, and the annual value derived only from local sources during high-wind conditions, more characteristic of its background pollution footprint. Finally, a combination of measurements from air quality networks with few fixed reference stations and those from mobile sensors such as Pollutrack should be combined to ensure that no bias related to the spatial distribution of measurements is present in the temporal analysis.

## Figures and Tables

**Table 1:** Annual PM2.5 trends and correlation between measurement and linear fit from Airparif and Pollutrack data.

Trend for PM2.5 Per Year	Mean (corr.)	Median (corr.)	Most Probable (corr.)
Airparif PM2.5 trend, no wind speed selection	−4.8 ± 0.4% (0.96)	−4.9 ± 0.5% (0.95)	−5.1 ± 0.5% (0.95)
Pollutrack PM2.5 trend, no wind speed selection	−6.0 ± 1.4% (0.89)	−5.7 ± 2.0% (0.78)	−4.5 ± 1.7% (0.76)
Airparif PM2.5 trend, winds < 6 m·s^−1^ range	−4.8 ± 0.4% (0.96)	−4.8 ± 0.5% (0.96)	−4.8 ± 0.6% (0.94)
Pollutrack PM2.5 trend, winds < 6 m·s^−1^ range	−6.0 ± 1.2% (0.91)	−5.8 ± 1.8% (0.82)	−5.3 ± 2.1% (0.74)
Airparif PM2.5 trend, winds > 6 m·s^−1^	−4.1 ± 0.4% (0.95)	−4.2 ± 0.4% (0.95)	−4.1 ± 0.4% (0.95)
Pollutrack PM2.5 trend, winds > 6 m·s^−1^	−5.2 ± 1.8% (0.78)	−4.0 ± 1.2% (0.84)	−3.7 ± 1.0% (0.87)

## Data Availability

Weather data are available at https://cds.climate.copernicus.eu/datasets/reanalysis-era5-single-levels?tab=overview. Airparif data are available at https://data-airparif-asso.opendata.arcgis.com/search?tags=pm25, accessed on 24 July 2025. The Pollutrack data are the property of the Pollutrack company, and can be asked to the company for scientific purposes only.
